# Defining blood hematology reference values in female pig-tailed macaques (*Macaca nemestrina*) using the Isolation Forest algorithm

**DOI:** 10.1111/jmp.12723

**Published:** 2024-08

**Authors:** Daniel Kim, Abigail Derton, George Khalil, Yi Pan, Shanon Bachman, Kristen Kelley, Gerardo Garcίa-Lerma, Charles W. Dobard, Michele B. Daly

**Affiliations:** 1 Laboratory Branch, Division of HIV Prevention, National Center for HIV, Viral Hepatitis, STD, and TB Prevention, Centers for Disease Control and Prevention, Atlanta, Georgia, USA; 2 Quantitative Sciences Branch, Division of HIV Prevention, National Center for HIV, Viral Hepatitis, STD, and TB Prevention, Centers for Disease Control and Prevention, Atlanta, Georgia, USA; 3 Office of Informatics and Data Management, Division of HIV Prevention, National Center for HIV, Viral Hepatitis, STD, and TB Prevention, Centers for Disease Control and Prevention, Atlanta, Georgia, USA

**Keywords:** complete blood counts, drug toxicities, non-human primates

## Abstract

**Background:**

Pig-tailed macaques (PTMs) are commonly used as preclinical models to assess antiretroviral drugs for HIV prevention research. Drug toxicities and disease pathologies are often preceded by changes in blood hematology. To better assess the safety profile of pharmaceuticals, we defined normal ranges of hematological values in PTMs using an Isolation Forest (*i*Forest) algorithm.

**Methods:**

Eighteen female PTMs were evaluated. Blood was collected 1–24 times per animal for a total of 159 samples. Complete blood counts were performed, and *i*Forest was used to analyze the hematology data to detect outliers.

**Results:**

Median, IQR, and ranges were calculated for 13 hematology parameters. From all samples, 22 outliers were detected. These outliers were excluded from the reference index.

**Conclusions:**

Using *i*Forest, we defined a normal range for hematology parameters in female PTMs. This reference index can be a valuable tool for future studies evaluating drug toxicities in PTMs.

## 1 | INTRODUCTION

Macaque models are critical for evaluating pre-exposure prophylaxis (PrEP) modalities for preventing human immunodeficiency virus (HIV) infections. These models have provided proof-of-concept research for all currently approved PrEP regimens in humans, including daily oral emtricitabine (FTC)/tenofovir disoproxil fumarate, FTC/tenofovir alafenamide, and long-acting cabotegravir for PrEP.^1–3^ The translatability of macaque models for studying HIV pathogenesis and prevention is in part due to the close phylogeny and physiology shared between macaques and humans. In addition, simian immunodeficiency virus (SIV) and simian/human immunodeficiency virus (SHIV) infections closely mimic HIV infection and pathogenesis, including the depletion of systemic and mucosal CD4^+^ T cells, chronic immune activation, and progression to simian acquired immunodeficiency syndrome.^4–8^

Pig-tailed macaques (PTMs) (*Macaca nemestrina*) are the preferred species for modeling vaginal HIV infection due to shared features with the human female reproductive tract, and unlike rhesus macaques (*Macaca mulatta*), which are seasonal breeders, female PTMs experience a lunar menstrual cycle.^9,10^ Hormonal fluctuations associated with the menstrual cycle have been shown to impact SIV/SHIV susceptibility, which should be carefully considered when evaluating the efficacy of prevention products.^11,12^ In addition, the PTM model has been refined to better mimic high-risk sexual transmission using repeated low-dose vaginal SHIV exposures and co-infections with other sexually transmitted infections.^13–15^ The combination of extensive historical data and continued refinements make the PTM model extremely valuable for assessing the PrEP efficacy of novel HIV prevention products.^16,17^

Safety and efficacy evaluations are priorities in preclinical studies, and as long-acting PrEP products become more prevalent, these studies can require lengthy assessments. Blood hematology is a common gauge of safety, particularly as it relates to drug toxicities and disease pathologies, which are often preceded by changes in complete blood counts (CBCs). As such, animal research facilities monitor the CBCs of animals to appraise health and aid illness diagnosis. Previous studies have reported baseline blood hematology values for rhesus macaques, Japanese macaques (*Macaca fuscata*), cynomolgus monkeys (*Macaca fascicularis*), and northern PTMs (*Macaca leonina*).^18–22^ However, published hematology information for southern PTMs (*Macaca nemestrina*) is limited to wild-caught or infant animals.^23,24^ One recent study published values for a single adult PTM, but this analysis was limited to white blood cells (WBC) and did not include subsets such as lymphocytes (LY), monocytes (MO), or granulocytes (GR).^25^

Thus, we aimed to characterize the hematology of female PTMs, as these animals are important for preclinical HIV research, and defining baseline ranges for healthy animals is imperative for understanding study outcomes. To create a reference index of hematology parameters, we evaluated a large sample set consisting of 159 CBCs from 18 female PTMs. We identified statistical outliers in our data set using the Isolation Forest (*i*Forest) model, which is an unsupervised algorithm that builds an ensemble of decision trees (*i*Trees) that repeatedly segment data using randomly selected features (i.e. hematology parameters) and split values.^26^ The assumption is that outliers will be isolated in fewer segmentations compared to normal data points. Using the *i*Forest data science method, we have established a reference index of blood hematology parameters in female PTMs. This reference will be an asset for any preclinical research studies involving PTMs, specifically for those evaluating drug toxicities and other disease pathologies.

## 2 | MATERIALS AND METHODS

### 2.1 | Humane care guidelines

The authors confirm that the ethical policies of the journal, as noted on the journal’s author guidelines page, have been adhered to and the appropriate ethical review committee approval has been received. This research was conducted under a Centers for Disease Control and Prevention Institutional Animal Care and Use Committee-approved protocol in compliance with the Animal Welfare Act, PHS Policy, and other federal statutes and regulations relating to the use of animals in research. Animals were housed in an AAALAC International accredited facility that adheres to principles stated in the Guide for the Care and Use of Laboratory Animals policy.^27^

### 2.2 | Study animals

Eighteen female PTMs with a median [range] age of 9 [7–17] years and a median [range] weight of 6.72 [5.65–12.05] kg were evaluated. Animals were not undergoing any clinical or protocol-related treatments with antiretrovirals or other drugs except for sedatives required for procedures. Blood was collected between 1 and 24 times per individual animal for a total of 159 samples. All macaques were purpose-bred and confirmed negative against *Mycobacterium tuberculosis*, *Trichuris* spp., *Shigella*, *Campylobacter*, *Salmonella*, *Yersinia*, simian retroviruses (SRVs), SIV, and simian T-lymphotropic viruses by the vendors. The macaques were not screened for herpes B virus.

### 2.3 | Sample blood collection

Before blood collections, animals were sedated with Ketamine (10 mg/kg; Dechra Veterinary Products) administered intramuscularly and if needed, were boosted with Telazol (2–6 mg/kg; Zoetis) administered intramuscularly. Approximately 1 cc of femoral venous blood was drawn into BD Vacutainer^®^ EDTA Tubes, using ethylenediamine tetra acetic acid-potassium (EDTA-K_2_) as the anti-coagulant.

### 2.4 | Complete blood count

Whole blood samples were inverted eight times immediately before analysis. CBCs were performed with the Beckman Coulter AcT diff2 Hematology Analyzer in the ‘closed vial whole blood’ mode. Hematology parameters analyzed included WBC, (10^3^/μL), LY, (10^3^/μL), MO, (10^3^/μL), GR, (10^3^/μL), red blood cells (RBC, 10^6^/μL), hemoglobin (HGB, g/dL), hematocrit (HCT, %), mean corpuscular volume (MCV, fL), mean corpuscular hemoglobin (MCH, pg), mean corpuscular hemoglobin concentration (MCHC, g/dL), red cell distribution width (RDW, %), platelets (Plt, 10^3^/μL), and mean platelet volume (MPV, fL).

### 2.5 | Statistical analyses

Outliers were detected using the *i*Forest model, implementing the scikit-learn package (v1.0.2) in Python (v3.9.13).^28^ This algorithm builds an ensemble of decision trees, called *i*Trees, which consist of nodes and branches. The n_estimators parameter was tuned by identifying the point of convergence (n_estimators = 1000) in plots of the average path lengths against the number of *i*Trees for randomly selected sets of data points. The max_features parameter was chosen to equal the total number of hematology parameters (max_features = 13). Histograms of anomaly scores were examined to assess the presence of outliers, and the ‘auto’ contamination parameter was chosen. All statistical analyses were conducted in Python (v3.9.13) and R (v4.3.1).

## 3 | RESULTS

### 3.1 | Complete blood counts

A total of 159 CBCs were analyzed from a cohort of 18 female PTMs. As outlined in Table 1, blood was collected between 1 and 24 times from each animal, with a median [range] of 5 [1–24] collections per individual. Macaques had blood collected weekly for up to 24 weeks, with differing number of collections based on animal allocation for other study needs. Thirteen hematology parameters were analyzed.

### 3.2 | Outlier detection

Outliers were detected using the *i*Forest algorithm with the following settings: max features = 13, contamination = “auto”, and n_estimators = 1000. In total, 22 outliers from 10 of the 18 animals were detected from the initial 159 sample set (Table 1).

### 3.3 | Blood hematology reference index

**FIGURE 1:**
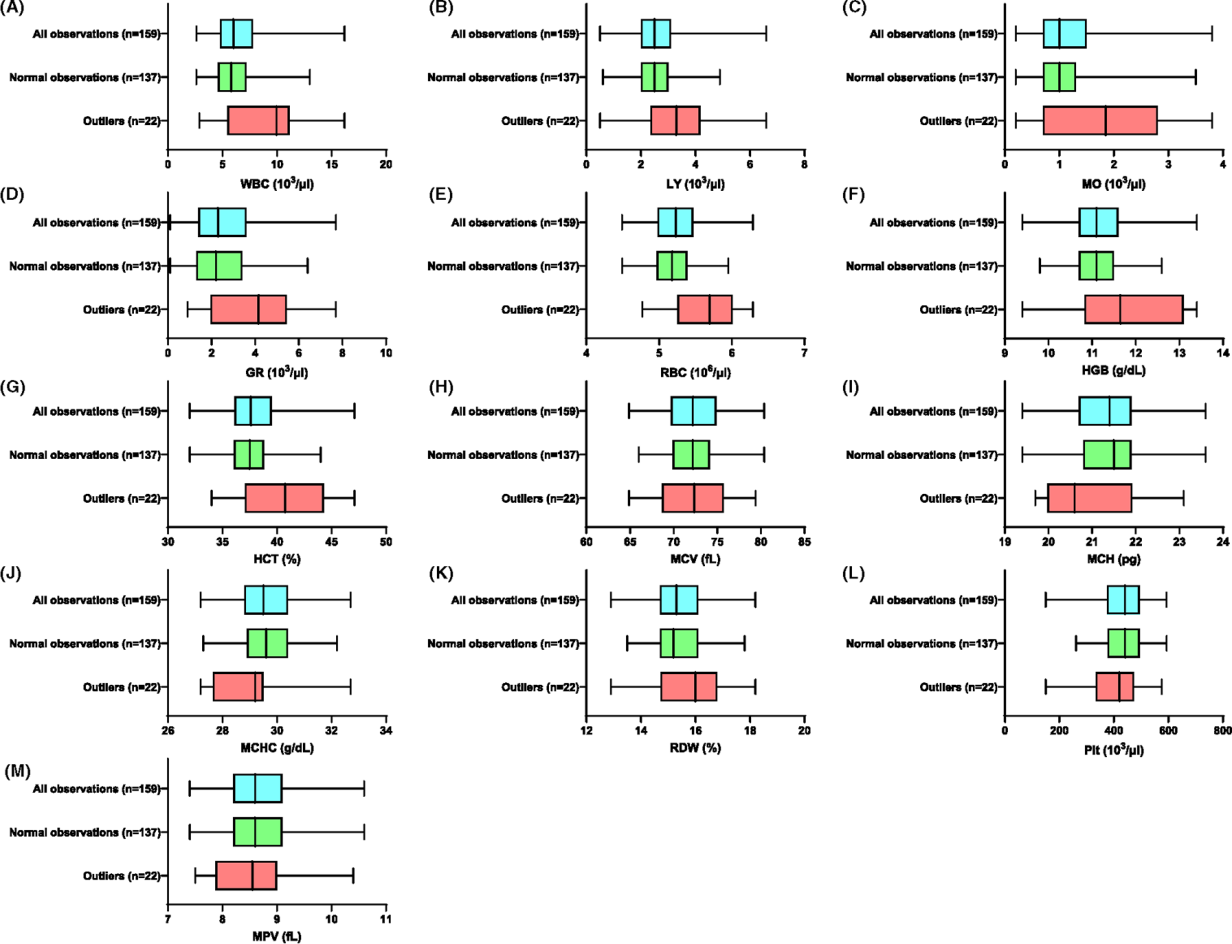
Distribution of hematology parameters. The data distribution of all observations (blue), normal observations (green), and outliers (red) shown for (A) white blood cells (WBC), (B) lymphocytes (LY), (C) monocytes (MO), (D) granulocytes (GR), (E) red blood cells (RBC), (F) hemoglobin (HGB), (G) hematocrit (HCT), (H) mean corpuscular volume (MCV), (I) mean corpuscular hemoglobin (MCH), (J) mean corpuscular hemoglobin concentration (MCHC), (K) red cell distribution width (RDW), (L) platelets (Plt), and (M) mean platelet volume (MPV). The box and whiskers of the plot represent the interquartile range (IQR), minimum, and maximum. The line in the box represents the median.

Each hematology parameter was plotted to visualize the effects of outlier removal on the distribution of the data set (Figure 1). As expected, the removal of outliers reduced the range, but the medians remained similar.

**FIGURE 2:**
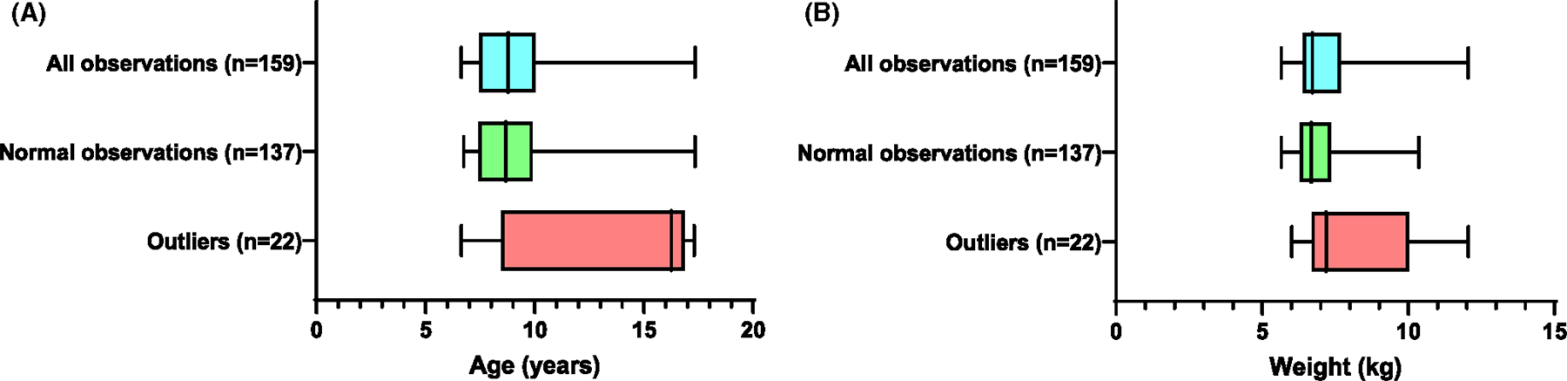
Macaque age and weight distribution. The data distribution of all observations (blue), normal observations (green), and outliers (red) is shown for (A) age and (B) weight. The box and whiskers plots show the interquartile range (IQR) and the minimum and maximum. The line in the box represents the median.

Age and weight are important factors to consider when allocating animals to research study groups.^29^ Therefore, we sought to understand if age and weight are determinants of hematological outliers. The median [range] age of animals that had outliers (*n* = 22) was 16.27 [6.63–17.31] years while the median [range] age of animals that did not have outliers (*n* = 137) was 8.67 [6.75–17.35] years. For weight, the median [range] of animals that had outliers (*n* = 22) was 7.19 [6.01–12.05] kg while the median [range] weight of animals that did not have outliers (*n* = 137) was 6.68 [5.65–10.63] kg (Figure 2).

After outliers were excluded from the data set, four PTMs from the initial 18 were completely excluded from further analysis (Table 1: BB125, Z15211, Z14140, and Z15336). A reference index of 13 blood hematology parameters was then created using 137 CBCs from 14 PTMs, with a median [range] of 7 [1–24] collections per animal (Table 2).

## 4 | DISCUSSION

Here, we report a reference index of blood hematology parameters in female PTMs, an important animal model for HIV research. These values provide an understanding of natural and expected variations in PTM hematology and can serve as a baseline for future studies evaluating drug safety and toxicities. The importance of these reference values was recently highlighted when clinical trials with the anti-HIV drug islatravir (ISL) were discontinued by the FDA after observing lymphopenia in the participants.^30^ Before its clinical hold, ISL was an attractive candidate for long-acting PrEP due to its high antiviral potency, long half-life, and predicted efficacy in macaque models.^31,32^ Unfortunately, the effect of ISL on LY was not fully investigated during early preclinical development. By providing an index of PTM reference values, our study may help to understand if the PTM model could detect lymphocyte toxicity due to ISL or other novel anti-HIV drugs prior to clinical advancement.

Hematology reference values have been reported for rhesus macaques (*Macaca mulatta*),^18,19^ Japanese macaques (*Macaca fuscata*),^20^ and cynomolgus macaques (*Macaca fascicularis*).^21^ Comparisons of their CBC values indicate many similarities among species and captivity status. However, there are some differences, such as WBCs, which were 2-fold greater in rhesus, Japanese, and cynomolgus macaques compared to PTMs. LYs and GRs were also 2-fold greater in rhesus and Japanese macaques than in PTMs. Interestingly, MO values were similar across all the studies. However, these variations may also have resulted from differences in the hematology analyzer used or other factors such as sedation and captivity status, making direct comparisons across studies difficult. Despite these factors, it is evident that the hematology of each species may have distinct and unpredictable characteristics, confirming the need for a reference index specific to PTMs.

For nonhuman primate research, the Animal Research: Reporting of In Vivo Experiments guidelines are followed when reporting study groups and results. During preclinical assessments of PrEP and other HIV treatments, these study groups are often randomized according to factors such as animal sex, age, and weight to control for innate differences in drug safety, pharmacokinetics, and efficacy.^33–35^ Thus, we sought to determine if age and/or weight impacted the likelihood an animal would have an outlier value in their hematology. The median age and weight of the outliers were higher than those of non-outliers, indicating that the majority of outliers were among the oldest and heaviest animals in our cohort, although their IQRs had some overlap with normal observations. This data reinforces the importance of age and weight-matched randomization for macaque research. Aging animals also have reduced regularity of hormonal cycles and low progesterone levels.^36,37^ We did not assess the menstrual cycle, but future studies evaluating the hematology in cycling female macaques could be beneficial to understand the effect of age and cycle phase on SHIV susceptibility.

To create a comprehensive reference index from this large data set, we employed the *i*Forest algorithm to detect outliers in place of classic statistical methods. We considered several other outlier detection algorithms, including Mahalanobis distance and one-class Support Vector Machine (SVM). The Mahalanobis distance method can identify outliers in multivariate normal data sets.^38^ However, 10 of the hematology features were not normally distributed, and thus, parametric methods such as Mahalanobis distance were not appropriate. One-class SVM is a non-parametric test that is appropriate for multivariate data. However, the *i*Forest method was selected over the one-class SVM due to faster run times and reduced sensitivity to outliers, which enabled the model to be trained on contaminated data (data containing both normal and outlier data points). Additionally, the *i*Forest method has several adjustable model parameters including contamination, max features, and n_estimators.

In this study, we analyzed 159 CBCs from 18 female PTMs, with a median of five blood collections per animal. One limitation of this data set is that each animal had different sampling frequencies, which may create a bias towards animals that were sampled more often. However, longitudinal sampling is also imperative in understanding how factors such as weight gain/loss, and aging can alter intra-animal variability. Additionally, other factors, such as infection, may induce immune responses that can cause significant changes in hematology. In this study, all animals were confirmed to be free of *Mycobacterium tuberculosis*, *Trichuris*, *Shigella*, *Campylobacter*, *Salmonella*, *Yersinia*, and SRVs through initial and routine health screenings. However, macaques were not screened for ubiquitous pathogens such as Herpes B virus or other active infections that have the potential to alter CBCs.^39^ Sedative medications and the frequency of sedation can also affect hematology. Previous studies have observed decreases in LY, HGB, HCT, and other parameters in rhesus macaques following sedations.^40^ All blood samples in this study were collected from anesthetized animals, providing consistency within the study samples tested. However, the effects of sedation should be considered when comparing these results to other studies that do not sedate animals for blood collection. Our results were obtained from the analysis of whole blood collected in K_2_ EDTA BD Vacutainer^®^ EDTA Tubes using the Beckman Coulter AcT diff2 Hematology Analyzer.^41^ Other analyzers and alternative blood diluents may yield varied results. Lastly, another potential limitation of our study was the use of the *i*Forest which has a tunable contamination parameter. This function requires a prior estimation of the percentage of outliers in the data. Due to the paucity of historical data on which to base these assumptions, the automatic estimation value was instead chosen for outlier analysis.

In conclusion, we defined a reference index of 13 hematology parameters from 137 CBCs from 14 female PTMs, using an *i*Forest algorithm to detect and remove outliers from the total data set. After removing outliers, we characterized the expected values and ranges for each hematology parameter. This established reference index will be useful for monitoring the health of PTMs and will provide important baseline data for future studies that utilize this animal model for preclinical research.

## Figures and Tables

**TABLE 1:** Sampling frequency and the number of outliers for each animal. One hundred and fifty-nine blood samples were collected from 18 female pig-tailed macaques for hematology analysis. The Isolation Forest algorithm was applied to detect statistical outliers.

Animal ID	Number of blood samples	Outliers
Z13076	24	0
Z15303	19	1
Z15228	19	0
Z15339	19	0
Z14009	17	4
Z13036	17	1
PDK2	11	4
Z15408	7	1
BB125	5	5
BB152	5	3
BB766	5	0
A10024	5	0
Z15211	1	1
Z14140	1	1
Z15336	1	1
Z14345	1	0
Z13099	1	0
Z15301	1	0
Total	159	22

**TABLE 2:** Reference values of blood hematology parameters in female pig-tailed macaques. Median [range] listed for the 13 blood hematology parameters. Outliers defined by *i*Forest were excluded.

Parameter (abbreviation, units)	Median [range] (*n* = 137)
White blood cells (WBC, 10^3^/μL)	5.80 [2.60–13.00]
Lymphocytes (LY, 10^3^/μL)	2.50 [0.60–4.90]
Monocytes (MO, 10^3^/μL)	1.00 [0.20–3.50]
Granulocytes (GR, 10^3^/μL)	2.20 [0.10–6.40]
Red blood cells (RBC, 10^6^/μL)	5.18 [4.49–5.95]
Hemoglobin (HGB, g/dL)	11.10 [9.80–12.60]
Hematocrit (HCT, %)	37.50 [32.00–44.00]
Mean corpuscular volume (MCV, fL)	72.20 [66.00–80.40]
Mean corpuscular hemoglobin (MCH, pg)	21.50 [19.40–23.60]
Mean corpuscular hemoglobin concentration (MCHC, g/dL)	29.60 [27.30–32.20]
Red cell distribution width (RDW, %)	15.20 [13.50–17.80]
Platelets (Plt, 10^3^/μL)	441.00 [261.00–593.00]
Mean platelet volume (MPV, fL)	8.60 [7.40–10.60]

## Data Availability

The data that support the findings of this study are available from the corresponding author upon request.
